# What We Have Learned so far From Single Cell Sequencing in Acute Kidney Injury

**DOI:** 10.3389/fphys.2022.933677

**Published:** 2022-06-08

**Authors:** Marc Buse, Marcus J. Moeller, Eleni Stamellou

**Affiliations:** Division of Nephrology and Clinical Immunology, RWTH Aachen University Hospital, Aachen, Germany

**Keywords:** tubular regeneration, acute kidney injury, single cell sequencing, chronic kidney disease, transcriptomics

## Abstract

Acute Kidney injury is a major clinical problem associated with increased morbidity and mortality. Despite, intensive research the clinical outcome remains poor and apart from supportive therapy no other specific therapy exists. Single cell technologies have enabled us to get deeper insights into the transcriptome of individual cells in complex tissues like the kidney. With respect to kidney injury, this would allow us to better define the unique role of individual cell populations in the pathophysiology of acute kidney injury and progression to chronic kidney disease. In this mini review, we would like to give an overview and discuss the current major findings in the field of acute kidney injury through Single-Cell technologies.

## Introduction

Acute kidney injury (AKI) is a major clinical problem and one of the most serious complications in hospitalized patients. It affects about two percent of all hospitalized patients and up to 35–57% patients in intensive care unit (Liangos et al., 2006; Ostermann and Chang, 2007; Bagshaw et al., 2008; Hoste et al., 2015). Additionally, its incidence has increased substantially over the last years due to the growing ageing population and increased prevalence of comorbidities such as diabetes or obesity (Susantitaphong et al., 2013; Hoste et al., 2015).

It is associated with high costs, prolonged hospital stays, and most important with a higher mortality (Hoste et al., 2015). Furthermore, acute kidney injury increases the risk of developing a chronic kidney disease (CKD) and end stage-renal-disease (Coca et al., 2012).

Despite a better understanding of the pathophysiology of AKI over the last years, still no specific therapy, apart from supportive measurements and dialysis, exist. Therefore, there is an urgent unmet need to better diagnose and treat AKI.

### Pathophysiology

AKI is a heterogeneous disease with various causes mainly including transient ischemia and/or toxic injury. Acute tubular injury accounts for the most common intrinsic cause for AKI. The main site of injury is the proximal tubule due to its high workload and energy demand. Upon injury, an intratubular subpopulation of proximal epithelial cells proliferates and restores tubular integrity. However, the origin of these cells still remains a controversy and so far, two hypotheses exist. One argues for the existence of a stable progenitor population (Sagrinati et al., 2006; Lazzeri et al., 2007; Ronconi et al., 2009; Angelotti et al., 2012) and the second supports that any tubular cell can adapt a transient regenerative phenotype as a common reaction to injury (Smeets et al., 2013; Berger et al., 2014; Kusaba et al., 2014; Stamellou et al., 2021). Nevertheless, despite its strong regenerative capacity, the kidney does not always achieve its former integrity and function and incomplete recovery leads to persistent and progressive CKD. (Yang et al., 2010; Chawla and Kimmel, 2012; Liu et al., 2017). The remaining nephrons then have to carry a higher workload leading to pathological hyperfiltration, hypertrophy and further fibrosis due to secondary glomerulosclerosis, ending in a vicious circle.

### The New Area of Single Cell Sequencing

Since the appearance of Next-Generation-Sequencing, it has been become possible to get deeper insights into the transcriptome. Even though bulk-RNA-Sequencing was a powerful resource, enabling to understand diseases at molecular level, it is limited in that relevant cell-specific gene expression signatures may be lost within the integrated expression profiles of the other cell types in the sample. Techniques for high throughput RNA-sequencing of individual single cells, so called single cell RNA-sequencing (scRNA-seq), introduced in 2009, have allowed to dissect the genetic program of single cells at very high resolution, enabling detection of heterogeneity among individual cells and characterization of rare cell population. While the evolution to spatial gene expression or ATAC-Sequencing have enabled individual cell resolution incorporated with spatial information and exploration of epigenetic modifications (Hwang et al., 2018).

### Cell Sequencing in Acute Kidney Injury

In the last 2 years (2020/21), three studies appeared which investigated acute kidney injury using Single-Cell-Sequencing technologies (Kirita et al., 2020; Rudman-Melnick et al., 2020; Gerhardt et al., 2021) and one using spatial transcriptomics (Dixon et al., 2022). In all of these, the Ischemia-Reperfusion-model (IRI) was used as an injury model (Table 1).

**TABLE 1:** IRI and scRNA seq platforms.

	IRI-Time (min)	Type of IRI	Time Points	Sex	Age	scRNA Platform
Gerhard et al	18	bilateral	7 and 28 d	male	11–19 w	10X Genomics
Rudman-Melnick et al	30	unilateral	1, 2, 4, 7, 11, and 14 d	male	4 and 10 w	DropSeq
Kirita et al	18	bilateral	4 h, 12 h, 2 d, 14 d, 42 d	male	8–10 w	10X Genomics
Dixon et al	34	Bilateral	4 h, 12 h, 2 d, 6 w	female	8–10 w	10X Genomics Visium

h, denotes hours; d, denotes days and w, denotes weeks.

The first study appeared in 2020 from Humphrey’s group (Kirita et al., 2020). In this study, single cell profiles were generated 4 h, 12 h, 2 days, 14 days, 6 weeks after injury in C57BL/6J wildtype mice. Early after induction of the injury (4 and 12 h), the authors could identify different clusters which contain healthy and injured proximal tubular cells (PTs) and were able to annotate them according to their origin (injured S1/2, injured S3) and the magnitude of injury (severe injured vs injured S1/2, S3). All injured clusters upregulated KIM-1 and share similarities with each other as well as with healthy PTs. Severe injured PT upregulated Keratin 20 and genes encoding heat shock proteins, proposing a stronger damage to these cells, and showed a cell cycle arrest. Interestingly the injured and severely injured clusters upregulated the myc gene, which encodes c-Myc and plays a role in cell cycle progression.

The injured and severely injured clusters almost disappeared 2 days after AKI, while another cluster which exhibited upregulation of cell cycle genes (i.e Top2a) appeared. Since this cluster had the highest proportion of proliferating cells, it was annotated as “repairing PT” cluster. In addition, by 2 days a new distinct cell cluster appeared, reaching almost 30% of all PTs by 14 days. These cells shared a unique transcriptional profile, characterized by the down-regulation of terminal differentiation markers and the up-regulation of a distinct set of genes i. e Vcam1, Sema5a, Dcdc2. This cluster was annotated as ‘failed repair proximal tubule cells. Gene set enrichment analysis revealed pathways related to inflammation. Immunofluorescence staining could confirm the presence of these cells in further injury models (folic acid nephropathy) and in human kidney allografts.

Finally, the authors investigated the intercellular communication within the kidney by performing ligand-receptor analysis and particularly they quantified Ccl2-Ccr2 signaling across all time points. Ccl2 and its receptor Ccr2 are important in the pathophysiology of AKI by recruiting T-cells and monocytes (Xu et al., 2019). Fibroblasts and endothelial cells signaled leucocytes first, followed by leucocyte-leucocyte signaling, whereas failed repair cells appeared at last to induce an increased signal.

Next, Gerhardt et al. from McMahon group, employed genetic labeling strategies focused on PTs using single nuclei RNA sequencing (Gerhardt et al., 2021). In this study mice were sacrificed 7 days (designated as early time point) and 28 days (designated as late time point) after induction of the injury. Based on the notion that keratin 20 represent an injury marker of PTs, in order to enable the isolation and tracing of Krt20^+^ cells, the authors generated a *Krt20* mouse line (Liu et al., 2017), which enabled irreversible tamoxifen-dependent labeling of nuclei in injured PTs.

Analysis revealed 7PT clusters which appear in both the control and Ischemia-reperfusion (IR) dataset and 5 clusters appearing only in IRI, annotated by the authors as IRI-clusters. Within these IRI-clusters there were two clusters which emerge mainly during the early time point (7 days) after AKI. One of these clusters exhibits exclusively a strong upregulation of cell-cycling genes like Mki67 and Top2a indicating a proliferative response to ischemia, similar as observed by Kirita et al. (Kirita et al., 2020). During the late time point these proliferative cells almost disappeared and another cluster related to inflammation and fibrosis appeared. These cells upregulated Pdgfd, Kcnip4, Vcam1 and Ccl2, all known markers for fibrosis and inflammation (Seron et al., 1991; Ostendorf et al., 2012; Kirita et al., 2020), while pathway analysis showed activation of AP-1 and NF-kB pathways, both pathways that have been identified previously to play an important role in driving kidney fibrosis after AKI (Liu et al., 2014; Ferenbach and Bonventre, 2015; Nakagawa et al., 2016; Kitani et al., 2022). Based on that, these cells were named maladaptive PTs. Next, in order to decipher the origin of these cells, the authors used a *Ki67* mouse line, which enabled tamoxifen-dependent labeling of proliferating cells. They assumed that these cells arise from proliferating cells which failed to repair. Intriguingly 89% of the maladaptive cells in the cortico-medullary boundary were GFP positive, whereas in the cortex only 27% of the maladaptive cells were GFP positive. Based on these results, the authors proposed that most cortical Vcam1^+^/*Ccl2* ^+^ cells either originate from cells that were injured during the initial IRI, but did not initiate replication or show obvious injury responses at this time, or from a secondary spread of the injury within the cortico-medullary boundary to the cortex, presumptively by paracrine signaling.

In the study of Rudman-Melnick et al. from Potter’s group, single cell profiles were generated 1, 2, 4, 7, 11 and 14 days after injury in Swiss-Webster (CFW) mice (Rudman-Melnick et al., 2020). Again, a cluster exhibiting a strong upregulation of cell-cycling could be identified, as observed in the above discussed studies (Kirita et al., 2020; Gerhardt et al., 2021). In addition, they identified a novel cell phenotype named as “mixed identity cells”, as these cells expressed ectopic markers of different cell types, i. e Umod (marker of loop of Henle), Lrp2 (marker for PT) and Nephrin (podocyte marker), while they down-regulate terminal differentiation markers. This cluster appeared directly after induction of the injury and it was no longer present at day 7. Next the authors, try to describe the gene expression patterns of injured tubule cells. Injured tubule cells were characterized by the downregulation of terminal differentiation proximal tubular markers, suggesting a dedifferentiation-process. Parallelly, they observed in these cells an elevation of genes implicated in nephrogenesis, i. e Sox4, Cd24, Hes1, Puuf3f3 and Hox genes. They focused mainly on Sox4, a transcription factor mainly expressed in the developing kidney (Yu et al., 2012) and Cd24, which encodes for a cell-surface sialoglycoprotein expressed during nephrogenesis (Challen et al., 2004). By studying Sox4 expression pattern in the injured kidney, interestingly they found an opposite relationship of Sox4 to terminal differentiation proximal tubule markers, i. e when resolution of injury began, Sox4 returned gradually to its original expression level and differentiation markers were upregulated, suggesting that Sox4 expression labels proximal tubule dedifferentiation. However, this was not the case in a subpopulation cell with a proinflammatory and profibrotic behavior, which showed prolonged elevated expression of Sox4 and no differentiation into PT. Furthermore, they observed that despite Cd24 is elevated in injured proximal tubule cells, its upregulation was more prominent in injured distal tubule. In addition, Spp1, which encodes secreted phophoprotein 1, and Cytokeratins (Krt7, Krt8, Krt18), both implicated in several kidney pathologies, among them renal cell carcinoma (RCC), found also to increase immediately after induction of the injury and remained upregulated till day 4, when they lowered to the normal levels. While later (by day 4), genes related to fibrosis, i. e Vim and Col18a1 upregulated in both injured and mixed identity cells. Finally, the authors compare the regenerative capacity of young to older mice (10 weeks) and they proposed an increased maladaptive response, estimated by a sustained expression of Sox4/Cd24a and missed upregulation of differentiation markers.

Dixon et al. from Humprhey’s group applied spatial transcriptomics during AKI and repair (Dixon et al., 2022). In this study, tissues were collected at early acute (4 and 12 h), early (2 days) and late (6 weeks) time points, while for the first time female C57BL6/J mice were used. The authors could identify several patterns of gene expression during injury and repair, i. e up-regulation of injury markers and down-regulation of proximal tubular markers. While, genes implicated in fibrosis or inflammation were rather upregulated only at late time points. Furthermore, they observed increased T cell and macrophage interactions with injured proximal tubular cells later on the course of injury retained up to 6 weeks, suggesting ongoing injury and inflammation. This work provides some additional findings regarding the molecular evidence and the cellular crosstalk of AKI to CKD transition.

## Discussion

Here, we describe the findings of four recently published works on AKI, through which it became possible to define better the cells implicated in the pathophysiology of AKI. Kirita et al. (Kirita et al., 2020) and Gerhardt et al. (Gerhardt et al., 2021) both describe a tubule cell population that fails to repair named either as failed-repair or maladaptive. These cells appear late in the course of injury and are characterized by the up-regulation of a new distinct set of genes, i. e Vcam-1, Ccl2, Pdgfd and by the down-regulation of terminal differentiation markers. According to the authors these cells are acquiring a proinflammatory and profibrotic phenotype, and it was suggested that they are implicated in the progression to CKD. Additionally, Rudman-Melnick et al. described a cell subpopulation with a proinflammatory and profibrotic phenotype, characterized by the prolonged elevated expression of Sox4 and Cd24, both genes associated with nephrogenesis and a later up-regulation of Vimentin. Interestingly most of the above-mentioned genes have been associated with a distinct proximal cell tubule phenotype, known as scattered tubular cells. Scattered tubular cells were firstly described by Lindgren et al. as a distinct cell subpopulation of cells acquiring a unique phenotype; smaller, with less cytoplasm, fewer mitochondria and less pronounced brush border (Lindgren et al., 2011). These cells are characterized by the expression of Cd24, Cd133, Vimentin, Vcam-1, Kim-1 and several other marker proteins (Sagrinati et al., 2006; Smeets et al., 2013; Kusaba et al., 2014). While, additionally they show down-regulation of terminal differentiation proximal tubule markers. However, as already mentioned above, there is a disagreement whether they represent a pre-existing intratubular cell population or whether all surviving cells acquire an equivalent regenerative capacity through dedifferentiation (Kramann et al., 2015; Stamellou et al., 2021).

To our opinion, the findings from the above discussed studies even though they do not exclude the existence of an intratubular progenitor population, they demonstrate that these cells rather represent damaged epithelial cells that have dedifferentiated and lost their epithelial characteristics than progenitor cells. If the highly cycling cells identified in all three studies represent progenitor cells remains still unclear and has still to be elucidated.

Finally, the study from Dixon et al. re-introduces a variable that is often disregarded which is the sex of the studied model. Animal models have consistently demonstrated that female sex is protective in the development of AKI after ischemia-reperfusion injury (Wyatt et al., 2016; Hosszu et al., 2020). Dixon et al. confirmed previous observations that there is a difference in male/female regarding the time of ischemia to induce similar extent of injury and identified some sex-specific differentially expressed genes.

Overall, we believe that these studies contribute significantly to solving the puzzle around AKI and AKI to CKD transition enabling us to understand the role of individual cells, with the overall aim to develop new strategies to treat AKI and prevent progression to CKD.
